# Glomerular Filtration Rate Assessment in Children

**DOI:** 10.3390/children9121995

**Published:** 2022-12-19

**Authors:** Sonja Golob Jančič, Mirjam Močnik, Nataša Marčun Varda

**Affiliations:** 1 Department of Paediatrics, University Medical Centre Maribor, Ljubljanska 5, 2000 Maribor, Slovenia; 2 Faculty of Medicine, University Medical Center Maribor, Taborska 8, 2000 Maribor, Slovenia

**Keywords:** glomerular filtration rate, assessment, children, chronic kidney disease

## Abstract

Glomerular filtration rate (GFR) measurement is a key tool for determining the degree of chronic kidney disease. The assessment of GFR is even more challenging in children than in adults with more variables in the equation than race and sex. Monitoring the progress of the kidney disease can therefore be difficult as in the initial stages of a decline in kidney function, there are no clinical signs. Due to children’s growth and development, changes in muscle mass and growth impair GFR estimation based solely on serum creatinine values. More invasive methods of GFR measurement are more reliable, but techniques using ionising agents, requiring large volume blood samples or timed voiding, have limited application in children. This paper reviews the methods of measuring and determining glomerular filtration rate and kidney function in children.

## 1. Introduction

Chronic kidney disease (CKD) is a growing problem in adults, but it is also important in children, although it occurs significantly less often. Research estimates the prevalence to be 74.7 cases per million children [1]. Children with CKD have risk factors for greater morbidity, mortality and poorer quality of life, and in a significant proportion of children, kidney failure occurs by the age of 20 [2]. CKD is defined as a structural or functional impairment of the kidney that persists for more than three months, regardless of cause [2]. Renal impairment progresses throughout life, often leading to end-stage renal disease (ESRD). The stages of CKD are defined as a decline in GFR according to the KDIGO guidelines [3]. CKD in childhood can have very different outcomes, depending on the cause [4]. Piepsz et al. have published GFR rates measured by Cr-EDTA clearance according to age [5]. (Table 1).

In children under two years of age, adult CKD rates are not used, as normal GFR values are lower than 60 mL/min/1.73 m^2^ [3]. Instead, GFR is monitored by measuring GFR. A decrease in GFR of one standard deviation (SD) from normal is defined as mild, two SD as moderate, and three SD as severe renal impairment. As a reference of normal values, the average measurements of GFR with inulin clearance in children under two years of age, as defined by Schwartz et al. [6], are used. For children younger than three months, the criterion of a structural change in the kidneys lasting more than three months does not apply either [3].

In the present article, an overview of the possible methods of GFR measurement for children and useful techniques in clinical practice are presented. In addition, the problems regarding this basic method in children are emphasised.

## 2. Glomerular Filtration Rate

GFR is currently the best estimate of kidney function. It reflects the excretory function of the kidneys, which is the sum of the GFR of all nephrons. GFR may decrease due to reduced blood flow or fewer functioning nephrons. With single or multiple determinations, the degree of kidney damage is determined; it is defined as acute or chronic, and the rate of progression of the kidney disease is determined [7].

GFR is influenced by the ultrafiltration pressure in the glomerular capillaries, capillary permeability and capillary surface area. Changes in any of these also result in a change in GFR [8].

The total GFR consists of the GFR of all individual nephrons. The reduced rate of the GFR in all nephrons at the same time is the result of a decrease in blood flow through the kidneys when the effective circulating volume is reduced (e.g., during dehydration). The cause can also be a disturbance in the outflow of urine due to a bilateral blockage of the flow of urine, e.g., in congenital urinary tract defects such as posterior urethral valves. In intrinsic kidney disease, the cause of the decrease in GFR is the deterioration of nephrons. When individual nephrons are damaged, initially the GFR and tubular resorption in the remaining healthy nephrons increase, so the overall decrease in the GFR is not proportional to the loss of kidney tissue, and the GFR may initially be normal despite kidney damage. As nephrons continue to deteriorate, the GFR also decreases. Because the kidneys have a large functional reserve, clinical signs and symptoms of uraemia appear only when the GFR is reduced to 5–10 mL/min [7].

## 3. Methods of Determining GFR

### 3.1. Clearance

Clearance is divided into kidney clearance and plasma clearance. The first is based on the measurement and calculation of the amount of filtered substance through the kidneys, which is why a constant concentration in the blood is required and, thus, more measurements from the urine. Plasma clearance is based on a single entry of a substance into the plasma and measurement of the concentration after a certain time, with which the elimination curve is measured and the excretion through the kidneys is calculated [9].

The most common method for measuring GFR relies on the concept of renal clearance [10]. In a healthy individual, GFR clears one volume of plasma in 20 min, and the entire amount of extracellular fluid in three hours [10]. A certain substance is thus removed from the plasma by GFR only after several passes of the plasma through the kidneys. If we know the concentration of substance x in the urine collected in 24 h, the excretion rate of substance x (in mL/min) and the concentration of substance x in the plasma, we can calculate the volume of plasma that is cleared of substance x by GFR in 24 h, i.e., the amount of glomerular filtration. The clearance of substance x (Cx) is calculated using the formula:

$$Cx=\frac{Ux\times V}{Px}$$

where V is the volume of urine, Ux is the concentration of substance x in the urine and Px is the concentration of substance x in plasma. The clearance is expressed in mL/min. If substance x is freely filtered across the glomeruli, is not secreted in the tubules and is also not synthesised or metabolised in the kidney, the clearance of substance x is equal to the GFR [10].

In plasma clearance, a known amount (M) of an exogenous substance is injected intravenously, and by measuring the concentration in the blood after a certain period of time, the amount of the substance that has been excreted is calculated. GFR is defined as the ratio between the rate of secretion of a substance and the concentration of secretion in time (t):

$$GF=\frac{dM/dt}{c\left(t\right)}$$

**Figure 1:**
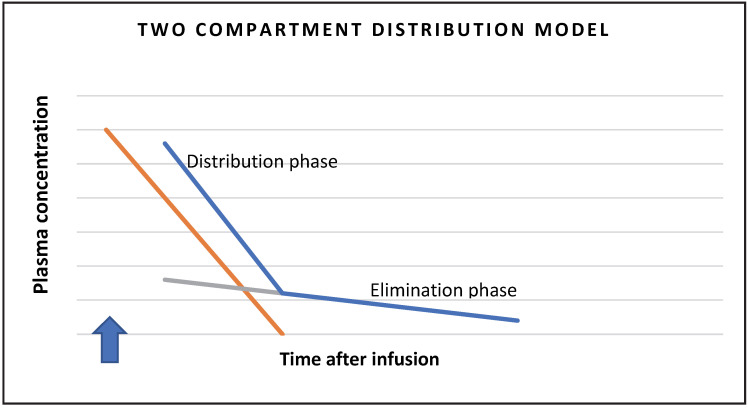
Two-compartment distribution model schematic presentation.

After a certain time, all the injected substance is excreted, so the amount of substance is equal to the area under the curve on the graph showing the concentration in the blood over time [9]. The lowering of the concentration in the blood depends initially on the distribution of the substance from the plasma to the distribution space (fast component or distribution phase), and then on the excretion via the kidneys (slow component or elimination phase). Therefore, we use a two-space model in the calculation schematically presented in Figure 1.

The normal value of GFR in adults is about 125 mL/min [7]. To compare the GFR of adults and children of different sizes, the latter must be standardised to a reference value. The most suitable reference value would be data on the weight of the kidneys, which is difficult to access but correlates well with the body surface area (BSA). GFR is therefore calculated on 1.73 m^2^ of the body surface. Calculation of GFR shows that children reach the GFR of adults at 6–24 months of age [10].

In adults, the BSA value is calculated according to the Du Bois formula [11]:

$$BSA\left(m^{2}\right)=weight{\left(kg\right)}^{0.425}\times height{\left(cm\right)}^{0.725}\times 0.007184$$

BSA in children and adolescents can be calculated according to the equation of Haycock et al. [12]:

$$BSA\left(m^{2}\right)=0.024265\times weight{\left(kg\right)}^{0.5378\;}\times height{\left(cm\right)}^{0.964}$$

In paediatric clinical practice, the Mosteller formula is most often used [13]:

$$BSA\left(m^{2}\right)=\sqrt{\frac{Height\left(cm\right)\times Weight\;\left(kg\right)}{3600}}$$

If non-glomerular excretion is negligible for the substance used, the clearance of the substance is equal to GFR. We can measure clearances of endogenous substances, such as creatinine, and exogenous substances, which are injected into a vein for this purpose, such as radioisotopes ^51^chromium-ethylenediaminetetraacetic acid (^51^Cr-EDTA), ^125^iodine-iotalatamate, ^99^Tc-diethylenetriaminoacetic acid (^99m^Tc-DMSA) or non-radioactive inulin and iohexol [10].

### 3.2. Creatinine Clearance

Creatinine clearance has established itself as a measure of GFR mainly due to the determination of creatinine, which is one of the oldest and most common laboratory tests. The determination is also easier than the measurement of the clearance of exogenous substances, and is more affordable. Creatinine is an endogenous product of creatine and phosphocreatine metabolism in muscles and reflects muscle mass. The concentration of creatinine in the blood is fairly constant. Creatinine is filtered in the glomeruli and secreted in the proximal tubules. Non-glomerular excretion is negligible in healthy individuals, but in patients with advanced CKD, this proportion may be higher resulting in falsely higher GFR values. Cimetidine can be added for research purposes, as it occupies the receptors for creatinine in the tubules and prevents tubular secretion [10].

For the examination, a 24-h urine collection and the plasma creatinine value, as well as the subject’s weight and height, are required. If the examination is carried out in children who cannot hold urine, a urinary catheter must be inserted.

### 3.3. Inulin Clearance

Renal clearance of inulin is the gold standard for determining the GFR in children and adults. Inulin does not bind to plasma proteins and is freely filtered through the glomerular membrane. The classic form of measurement of inulin clearance requires an intravenous injection of an initial dose of inulin followed by a steady 2- to 3-h infusion of inulin to achieve a constant plasma concentration for the required 45 min [14]. Urine is then collected for 10–20 min, which is more demanding in younger children and children with a neurogenic bladder, non-functional micturition or vesicoureteral reflux.

This method of measurement is still the gold standard for determining GFR, but it is technically very demanding. In addition, not all laboratories are equipped to measure the concentration of inulin, and the procedure is expensive [8].

The plasma clearance of inulin (C_in_) can also be determined by taking blood and determining the serum concentration of inulin (S_in_) after establishing the equilibrium state of inulin in the plasma, taking into account the speed (H) and concentration of inulin in the infusion (I_in_) [15]:

$$C_{in}=I_{in}\times \frac{H}{S_{in}}$$

units: C_in_—mL/min; I_in_—mg/mL; S_in_—mg/mL; H—mL/min.

By determining the plasma clearance of inulin, the procedure is somewhat simplified, as urine collection is not required. Swinkels et al. tried the latter in children with a single administration of inulin and as little blood sampling as possible. With blood sampling, we determine the elimination curve, which can be divided according to the two-space model into the distribution phase, when inulin is distributed from the intravascular to the interstitial space, and the elimination phase, when the concentration in the blood decreases at the expense of renal excretion [13]. They found the optimal number of withdrawals to be seven, at 0 min, 10 min, 20 min and 30 min, and then 65 min, 120 min and 240 min, to best describe the elimination curve of the two-compartment model [16].

### 3.4. Iohexol Clearance

Iohexol is a nonionic contrast agent with low osmolarity. It is used intravenously in much higher doses for radiological diagnosis and interventions even in the presence of kidney disease, as it is not absorbed, metabolised or excreted by the kidneys. The molecular weight of iohexol is 821 Da and it is a cheap and standardised preparation that has been used in medicine for a long time. Caution is required only in patients who are allergic to iodine. To measure GFR with iohexol, plasma clearance of the substance is used [10]. It is excreted completely unchanged in the urine and, due to its smaller molecular weight, is distributed throughout the body more quickly than inulin. In Scandinavia [17] and the USA, there were no significant side effects when GFR was determined with iohexol [18].

In the initial research, Schwartz et al. showed that the GFR measurement with iohexol is effective after a single dose of iohexol with nine blood samples taken at 10, 20, 30, 60, 120, 180, 240 and 360 min [19]. They also found that the calculation of the two-space model correlates well even with four withdrawals (r = 0.999). When considering the one-space model, we use the Brochner-Mortensen equation for correction, which is similar to the ^51^Cr-EDTA measurement. Sufficient time must elapse from intake to estimation of GFR using a unicompartmental model, especially in low GFR or preterm infants [10]. Attempts have also been made to collect a drop of blood on filter paper for later determination of the concentration of iohexol in the blood, which is relatively accurate [20].

There is currently no standardised procedure for measuring GFR with iohexol. However, there are several studies of measurements using different protocols and different numbers of samples in children; in some, it was also compared with renal creatinine clearance [19,21,22,23,24,25,26,27]. A comparison between the plasma clearance of iohexol and the renal clearance of inulin showed a good correlation between the two measurements, with four blood samples collected in the second phase [28].

Several studies and commentaries have been published regarding the use of iohexol as a means of measuring GFR in children, confirming its usefulness in determining the degree of CKD. Currently, there are discussions regarding the standardisation of the method and the number and method of sampling for all children with CKD [29].

### 3.5. ^51^Cr-EDTA and ^99m^Tc—DMSA Clearance

These radioactive molecules have been used for a relatively long time to determine GFR. EDTA and DMSA are similar binding molecules that carry a radioactive element suitable for measurement. They bind poorly to plasma proteins and are mostly filtered through the kidneys. At appropriate doses, the radiation with ^51^Cr-EDTA is 0.6 mSv and the radiation with ^99m^Tc-DMSA is 0.05 mSv in an adult. Recently, they have been less available in Europe [10]. Renal clearance of both substances can be used, but plasma purification is usually preferred. The concentration can also be determined with a gamma camera in combination with or without blood sampling [30].

After the intravenous injection of the radiopharmaceutical, several blood samples are taken, and by measuring the radioactivity of the sample over time, the curve of its elimination rate is determined. The slope of the curve depends on the clearance of the radiopharmaceutical. Radiopharmaceuticals for determining GFR and effective plasma flow through the renal parenchyma are labelled with different radioisotopes; so, with nuclear medicine methods, both measurements can be performed simultaneously, and the filtration fraction (FF) calculated. FF is defined as the ratio between GFR and effective plasma flow through the renal parenchyma. Normally, it is 0.20–0.30 [7].

## 4. Glomerular Filtration Rate Estimation (eGFR)

The GFR estimate is based on a calculation using an endogenous marker such as creatinine or cystatin C. The calculations are based on population calculations and can be quite complex, but it is important that the method for the determination of the marker is the same as provided for the equation. In the past, the classic Jaffe reaction was mostly used to determine creatinine, but the result was affected by the presence of many substances (e.g., albumin, glucose, bilirubin, cephalosporins). However, with modifications of the method, its specificity has increased. Isotope dilution mass spectrometry has been adopted worldwide as the reference (definitive) method for determining serum creatinine. For better comparability between laboratories, an international standard for creatinine—SRM 967, prepared in 2004—enables traceability to this standard and is followed by most manufacturers of laboratory equipment. Only based on traceability to the standard can the serum creatinine concentration values of a certain laboratory be “trusted.” As an optional working method, laboratories were advised to use an enzyme reaction, which is specific but more expensive; hence, the modified Jaffe reaction, which is adequate in the case of traceability to the standard, is still mainly used. Such an agreement was also adopted in Slovenia and has been gradually implemented in practice since 2009 [7].

### 4.1. Cockcroft-Gault Equation

The Cockcroft–Gault (CG) equation was based on a 1973 study of 249 men with a measured creatinine clearance of 30–130 mL/min. It is used to estimate creatinine clearance (Clcreat), but not GFR. When using this equation, age, sex, body weight and serum creatinine (S-creatinine) concentration are considered. For women who were not included in the research, a correction factor was introduced based on the fact that in women, due to their lower body weight, the production of creatinine in the body is 15% lower than in men.

$$Clcreat=\frac{\left(140-age\right)\times weight\;}{0.814\times Screat}\times \left(0.85\;\mathrm{for}\;\mathrm{women}\right)$$

units: Clcreat—mL/min; age—years; body weight—kg, S-creatinine—μmol/L. The result must then be converted to 1.73 m^2^ of body surface area.

The equation systematically overestimates GFR and additionally overestimates GFR in vegetarians, obese and oedematous patients and people on low-protein diets. GFR is underestimated in young people and patients with CKD [31]. Because of the conversion to standard body surface area, the calculated creatinine clearance in people with a large or small body surface area is therefore incorrect. In the past, all recommendations on the adjustment of drug doses in patients with reduced creatinine clearance were based on the CG equation. In recent years, it has increasingly been replaced in practice by the MDRD (Modification of Diet in Renal Disease, MDRD) research equation and the CKD-EPI (chronic kidney disease epidemiology collaboration) research [7]. The CG equation was developed for adults and is not suitable for children under the age of 12, as it only takes body weight into account and has even greater errors in children. Its validation was not successful in children under 18 years of age [32].

### 4.2. The Four-Variable MDRD Equation

This equation is a by-product of a large 1999 study on the impact of diet on CKD, which included 1628 patients. In addition to the creatinine value, it also considers the subject’s gender, age and ethnicity. The eGFR is expressed for an average body surface of 1.73 m^2^. It was originally intended for the Jaffe method of creatinine determination, so in 2005 the factor was updated from 186 to 175 [31].

$$eGFR=175\times {\left(\frac{Screat}{88.4}\right)}^{-1,154}\times {\left(age\right)}^{-0.203}\times \left(0.742\;for\;women\right)\times \left(1.210\;for\;African\;Americans\right)$$

units = eGFR—mL/min/1.73 m^2^; serum creatinine (Screatine)—μmol/L); age—years.

The equation is often used for adults and is less accurate in younger patients and in patients who do not have CKD, such as younger patients with type 1 diabetes and potential kidney donors [31].

### 4.3. The CKD-EPI Equation

The CKD-EPI equation was created in 2009 and is based on the same variables as the MDRD equation but is more complex. The study included CKD patients, diabetic patients, kidney transplant patients and healthy people, improving the accuracy of the GFR estimation in these human populations. When determining eGFR CKD-EPI, eGFR values below 60 mL/min/1.73 m^2^ are as accurate as eGFR MDRD, but above this range, eGFR CKD-EPI is more accurate. Using the CKD-EPI equation, compared to the MDRD equation, the proportion of people with eGFR < 60 mL/min/1.73 m^2^ is reduced by 10%, especially in those under 60 years of age, and thus as many as 35% fewer people have eGFR < 60 mL/min/1.73 m^2^. Recently, therefore, instead of the MDRD equation, the CKD-EPI equation [7] has been recommended for eGFR. In Slovenia, the CKD-EPI equation has been recommended for laboratory use since 2009, but it is not validated for children under 18 years of age [32].

### 4.4. The Schwartz Equation

This equation, which is also used in children, includes body height, which is used to predict muscle mass and thus also estimates creatinine excretion from muscles. The value of eGFR is given in mL/min/1.73 m^2^ and includes the factor k, which depends on age and is 0.33 for prematurely born children up to 1 year of age, and 0.44 for full-term children. From one to thirteen years, the factor is 0.55 for both sexes, and for adolescents, it is 0.70 for men and 0.55 for women [33].

$$eGFR=k\times \frac{height}{Screat/88.4}$$

units: eGFR—mL/min/1.73 m^2^; body height—cm; serum creatinine (Screat)—μmol/L.

The equation was designed to estimate GFR in children and is therefore the most widely used bedside equation for estimating GFR in children. Due to its reliance on serum creatinine, it is quite inaccurate in children with a low muscle mass, and is also significantly affected by protein intake. Compared with GFR measured with iohexol, this equation was found to overestimate GFR [18].

Based on a more recent CKiD study, an updated Schwartz equation was constructed in 2009, which also considers the enzymatic method of determining creatinine and, in addition to serum creatinine, includes the serum value of urea, cystatin C and gender [34]:

$$\mathrm{eGFR}=39.1\times {\left(\frac{height}{Screat\;/88.4}\right)}^{0.516}\times {\left(\frac{1.8}{ScistC}\right)}^{0.294}\times {\left(\frac{30}{Surea}\right)}^{0.169}\times {\left(1.099\right)}^{male}\times {\left(\frac{height}{1.4}\right)}^{0.188}$$

units: eGFR—mL/min/1.73 m^2^; body height—m; serum creatinine (Screat) (μmol/L); serum concentration of urea (Surea)—mg/dL; serum concentration of cystatin C (ScistC)—mg/L.

Since the latter is much more complicated, it was converted into a bedside equation for the sake of simplification:

$$eGFR=0.412\times \frac{height}{Screat}$$

if height is expressed in cm and serum creatinine in mg/dL, respectively:

$$eGFR=36.2\times \frac{height}{Screat}$$

if height is expressed in cm and serum creatinine in μmol/L.

The CKiD equation compared to the measured GFR with iohexol correlated with R = 0.85, and the mean error was −1.60 mL/min. The bedside version of the equation correlated with the measured GFR with a coefficient of R = 0.84 with a mean error of 1.75 mL/min. Both equations give very accurate GFR estimates in the range of 15–75 mL/min/1.73 m^2^ and probably even at a GFR above 90 mL/min/1.73 m^2^. Since the calculation was performed on a group of children with GFR up to 75 mL/min/1.73 m^2^, when calculating with these equations, >75 mL/min/1.73 m^2^ are given as higher values.

In 2012, the equation was corrected for the nephelometric method of measuring cystatin C [34]:

$$eGFR=39.8\times {\left(\frac{height}{Screat/88.4}\right)}^{0.456}\times {\left(\frac{1.8}{ScistC}\right)}^{0.418}\times {\left(\frac{30}{Surea}\right)}^{0.079}\times 1.076^{male}\times {\left(\frac{height}{1.4}\right)}^{0.179}$$

units: eGFR—mL/min/1.73 m^2^; body height—m; serum creatinine (Screat)—μmol/L; serum concentration of urea (Surea)—mg/dL; serum concentration of cystatin C (ScistC)—mg/L.

### 4.5. Cystatin C

Cystatin C is a protein from the group of cysteine protease inhibitors. It is produced in all nucleated cells at a constant rate and has a low molecular weight. It is found mainly in extracellular fluids, i.e., in blood, cerebrospinal fluid and seminal fluid. It is filtered in the glomeruli, and is not secreted in the tubules, but is completely resorbed and degraded in the proximal tubules. Therefore, it no longer passes into the bloodstream and is thus a suitable endogenous marker for GFR assessment. Unlike serum creatinine, cystatin C is not affected by age, sex, muscle mass, body composition, inflammatory processes or diet [7].

In the absence of renal disease, the serum concentration of cystatin C is increased in patients with liver disease, thyroid disease and during treatment with glucocorticoids [7]. Since it is broken down in the kidneys, it is not found in the urine and the calculation of renal clearance is not possible. The level of cystatin C in healthy adults is 0.8–1 mg/L [35], and research on samples from 291 children showed that children reach adult levels at the age of one year [36].

Several equations have been published to estimate GFR using the cystatin C level, and in some equations already mentioned, the serum cystatin C level is used together with the serum creatinine level to estimate GFR:

- CKiD-2012, Schwartz et al. [34]: $eGFR=70.69\times \left(ScistC^{-0.931}\right)$
- Zapitelli et al. [37]: $eGFR=75.94\times \left(ScistC^{-1.17}\right)$
- Filler et al. [38]: $eGFR=91.62\times \left(ScistC^{-1.123}\right)$

Units: serum level of cystatin C (ScistC)—mg/L.

According to the above equations, it is clear that in all cases, it is a mathematical derivation of the measured GFRs. The estimated GFR using cystatin C is more accurate than using creatinine in children younger than two years. The equations also differ due to the different times of formation and therefore there is no standardised determination of cystatin C [9].

A study was conducted to assess GFR in newborns, which also takes ultrasound-measured kidney volume into account [39]:

eGFR = [(Vol-T/BSA)/Scistc]/1.73

where: Vol-T—total kidney volume; BSA—body surface area; ScistC—serum level of cystatin C in mg/L.

## 5. Measurement and Estimation of Glomerular Filtration Rate According to Age

The basic characteristics of children is their growth and development, therefore not all the described methods are appropriate in every age.

Neonates, especially preterms, are unique in their physiological changes. Normal variations of serum creatinine and cystatin C should be considered. At birth, serum creatinine is elevated due to its placental passage during pregnancy, and does not properly reflect GFR. Therefore, serum creatinine does not present the best marker for GFR estimation after birth; however, its level might be useful in observing its dynamics—an additional rise or failure to fall appropriately might indicate an acute kidney injury, to which neonates are especially prone to [40]. On the contrary, cystatin C does not cross the placenta and seems to correspond to GFR better. However, at birth, the levels of cystatin C are also elevated, which is presumed to be due to the physiological lower GFR [40,41]. As the GFR physiologically increases, cystatin C levels start to fall and reach steady state at around 2 years of age, the same time when full GFR is achieved physiologically [36]. 

After that, the decision on the form of determination of GFR is based on the clinical state of the patient. Guidelines recommend the use of the creatinine-based updated Schwartz formula in children [40], which underestimates GFR in young adults. On the contrary, using the CKD-EPI formula, used more often in the adult population, can overestimate GFR in young adults [40]. Therefore, this age group also presents special consideration of which method to use considering muscle state and expected kidney function. Cystatin C-based equations or iohexol use might be considered in such clinical situations as well as in the general paediatric population when creatinine-based formulas provide borderline results and an additional measurement provides a tool for clinical decision-making.

## 6. Future Perspectives

Along with creatinine and cystatin C, other endogenous molecules are being researched. The measurement of an endogenous marker is preferred due to the less invasive and demanding procedure along with endogenous markers being always accessible in contrast to some exogenous markers, such as inulin. Thus, research into beta-trace protein and beta-2 microglobulin as endogenous markers of kidney function has increased. Beta-trace protein is synthesised mainly in the central nervous system and forms one of the principal constituents of the cerebrospinal fluid, where its concentration is the highest. Some of the protein also originates from the kidneys, genital organs and heart; it is subsequently metabolised by the liver and eliminated almost exclusively via the kidneys. It has been found to be an early marker of impaired kidney function with urinary excretion inversely related to GFR. It was increased when the GFR fell below 90 mL/min1.73 m^2^. It has been hypothesised that this is the consequence of the lower reabsorption capacity of the nephron for beta-trace protein. It also has a large molecular size, and it does not cross conventional haemodialysis membranes, which makes it a useful marker of residual renal function in dialysis patients [42]. 

Beta-2-microglobulin on the other hand is a small protein found on the surface membrane of nearly all nucleated cells. It is shed during membrane turnover and can be detected in various fluids with the highest concentrations in serum and synovial fluid. It is reabsorbed and catabolised in the proximal tubules. Elevated urine concentrations are indicative of tubular dysfunction, and are also linked to viral infections, inflammation and various types of malignancy. It does not cross the placental barrier and can therefore be used as a parameter of foetal renal function. Otherwise, it is unclear whether the determination of its concentration adds significant benefit in the paediatric population [42]. 

Other novel biomarkers of tubular injury, including neutrophil gelatinase-associated lipocalin (known as NGAL), kidney injury molecule-1, liver-type fatty acid binding protein, N-acetyl-β-(D)-glucosaminidase, interleukin-18 and urinary epidermal growth factor [43,44,45], which may enable the early detection of acute kidney injury before or in the absence of a change in GFR, are being researched. 

In addition, we hope that research will also continue in the fields of accurate, child-friendly GFR determination and early renal risk detection.

## 7. Conclusions

The value of GFR can be accurately determined by measuring GFR with exogenous substances, which are the gold standard. Recently, radioactive markers have been increasingly replaced by the non-ionising marker iohexol. 

In the clinical context, it is important to utilise a method that has good accuracy but is not too burdensome for patients of all ages. In our practice, we obtained diametrically different results of GFR estimation from creatinine or by measuring creatinine clearance from 24-h urine collection. In patients with persistent discrepancies in GFR measurements with CKD, we measured iohexol clearance. 

In our hospital, the GFR value is determined with iohexol in both children and adults, mostly with five blood samples, but protocols with a smaller number of samples are being developed. The measurement of GFR with iohexol is a welcome innovation in our clinic to assist in the assessment of the progression of CKD. 

The assessment of GFR based on the serum creatinine level has already been implemented in the laboratory system, and reference values are considered when determining serum cystatin C. Accurate determination with exogenous markers is especially important for assessing the progression of CKD in children, which, as a rule, does not show other clinical signs in the early stages. Further assessment of other potential, possibly endogenous markers of kidney function is needed.

## Figures and Tables

**Table 1:** Plasma 51Cr-EDTA clearance in normal infants and children. Data form [5].

Age (months)	Mean GFR ± SD (mL/min/1.73 m^2^)
<1.2	52.0 ± 9.0
1.2–3.6	61.7 ± 14.3
3.6–7.9	71.7 ± 13.9
7.9–12	82.6 ± 17.3
12–18	91.5 ± 17.8
18–24	94.5 ± 18.1
>24	104.4 ± 19.9

## Data Availability

Not applicable.
